# The mitochondrial and death receptor pathways involved in the thymocytes apoptosis induced by aflatoxin B_1_

**DOI:** 10.18632/oncotarget.7731

**Published:** 2016-02-25

**Authors:** Xi Peng, Zhengqiang Yu, Na Liang, Xiaofeng Chi, Xiaochong Li, Min Jiang, Jing Fang, Hengmin Cui, Weimin Lai, Yi Zhou, Shan Zhou

**Affiliations:** ^1^ Key Laboratory of Animal Diseases and Environmental Hazards of Sichuan Province, College of Veterinary Medicine, Sichuan Agricultural University, Chengdu, Sichuan, PR China

**Keywords:** aflatoxin B1, thymocytes, apoptosis, signaling pathways, broilers, Immunology and Microbiology Section, Immune response, Immunity

## Abstract

Aflatoxin B_1_ (AFB_1_) is a potent immunosuppressive agent in endotherms, which can be related to the up-regulated apoptosis of immune organs. In this study, we investigated the roles of the mitochondrial, death receptor, and endoplasmic reticulum pathways in Aflatoxin B_1_ induced thymocytes apoptosis. Chickens were fed an aflatoxin B_1_ containing diet (0.6 mg/kg AFB_1_) for 3 weeks. Our results showed that (1) AFB_1_ diet induced the decrease of T-cell subsets, morphological changes, and excessive apoptosis of thymus. (2) The excessive apoptosis involved the mitochondrial pathway (up-regulation of Bax, Bak, cytC and down-regulation of Bcl-2 and Bcl-xL) and death receptor pathway (up-regulation of FasL, Fas and FADD). (3) Oxidative stress, an apoptosis inducer, was confirmed in the thymus. In conclusion, this is the first study to demonstrate that mitochondrial and death receptor pathways involved in AFB_1_ induced thymocytes apoptosis in broilers.

## INTRODUCTION

Aflatoxins, a group of mutagenic compounds and a contaminant of many food sources, especially in some parts of Africa, Asia and Latin America. The naturally occurring aflatoxins are aflatoxin B_1_, B_2_, G_1_ and G2 (AFB_1_, AFB_2_, AFG_1_ and AFG_2_), in which AFB_1_ is the most abundant, toxic and carcinogenic [1-3]. Williams et al. [4] have estimated that 4.5 billion of the world's population is exposed to aflatoxins. In animals, these toxins also impair growth and are immunosuppressive. The latter effect is of increasing interest in human populations, because an increased tumors incidence probably is resulted from the poor immunity of the host [5, 6].

Secondary to the effect on liver, the immunosuppressive nature of AFB_1_ is the best documented area of its toxicity [7], but there are few studies on the relationship of aflatoxins and immunity in human. Previous researches showed that dietary AFB_1_ could decrease secretory IgA (sIgA) in saliva production, antibody response, percentage of CD3^+^, CD4^+^, CD8^+^, and CD19^+^ lymphocytes, and level of cytokines in peripheral blood in human [8-10]. In livestock and laboratory animals, the negative effects associated with AFB_1_ exposure on immune system include the decrease of relative weight of immune organs, T-cell subsets, cytokines, antibody titers, complement activity, and the increase incidence of lymphoid tissues injury [11-14]. Furthermore, several studies suggested that AFB_1_ treatment could induce oxidative stress, cell cycle arrest, excessive apoptosis, and mitochondria injury in lymphoid tissues [15-18]. These findings indicated that the injuries of immune organs might play critical roles in immunosuppression induced by AFB_1_ administration, but its mechanisms need to be further clarified.

Recently, it is well accepted that apoptosis in immunocytes is important for immunosuppressive function in human and various animal species *in vivo* and *in vitro* [19-24]. Chen et al. [17] showed that oxidative stress and apoptosis play key roles in AFB_1_ induced toxicity of immune organs. As we know that the mitochondria, death receptor, and endoplasmic reticulum mediated apoptotic pathways are the three key pathways in apoptosis [25]. However, the exact mechanism of AFB_1_ induced thymus apoptosis has not been elucidated. To address this, we use a broiler model to examine the roles of mitochondria, death receptors, and endoplasmic reticulum pathways in AFB_1_ induced toxicity of thymocytes. We analyzed the histological and ultra-structural changes of thymus, T cell subsets, mitochondrial membrane potential, percentage of apoptotic thymocytes, and relative expression of apoptosis-regulating genes. Taken together, our data reveal that the mitochondrial and death receptor pathways are involved in AFB_1_ induced thymocytes apoptosis.

## RESULTS

### Histopathological and ultrastructural analysis

**Figure 1:**
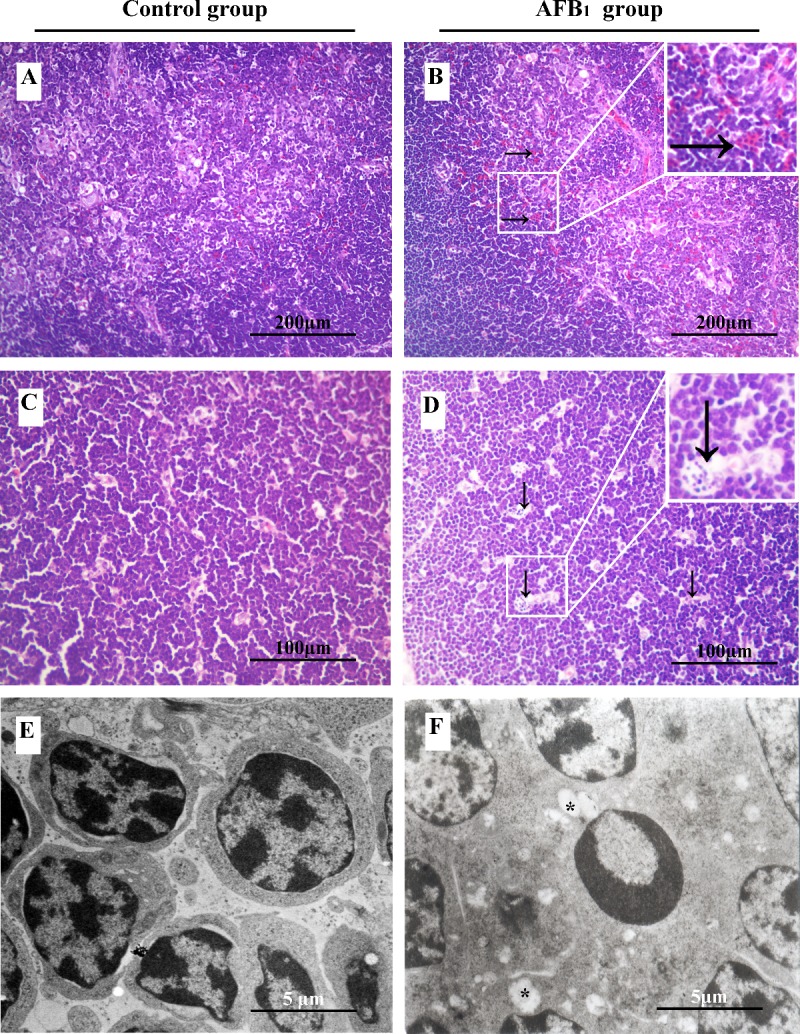
The impact of aflatoxin B_1_ exposure on thymus histopathology (congestion and nuclear debris) and ultra-structural pathology (mitochondria vacuole and chromatin margination) Histological assessment of H&E-stained thymus tissues from 21-day-old broilers exposed to AFB_1_ diet **B.** and **D.** and control diet **A.** and **C.**. Congestion in thymic medulla **↓.**, **D.** and increased number of nuclear debris **→.**, **B.**. Ultrastructural assessment of uranyl acetate and lead citrate stained thymus sections from the control **E.** and AFB_1_ group **F.**. Vacuolated mitochondria with degenerated cristae (*, F), and chromatin margination in lymphocytes.

In the control group, the morphology of the epithelial reticular cells was clear in the contex and medulla, and there were no obvious histopathological changes in the thymus (Figure 1A, 1C). When compared with the control group, congestion, degenerated reticulocytes and increased nuclear debris were observed in the AFB_1_ group. The congestion showed as relatively increased capillaries and expanded vessels containing many blood cells, the degenerated reticulocytes had unclear nuclear shape and were surrounded by more debris (Figure 1B, 1D). The incidence of congestion in medulla and increased nuclear fragmentation of thymus was showed in Table 2.

**Table 1:** A list of oligonucleotides used as primers in qRT-PCR analysis of gene expression in chicken thymocytes

Gene symbol	RefSeq mRNA number	Forward primers	Reverse primers	Amplicon length (bp)
GSH-Px	NM001277853	TTGTAAACATCAGGGGCAAA	TGGGCCAAGATCTTTCTGTAA	140
CuZn-SOD	NM205064	CGCAGGTGCTCACTTTAATCC	CTATTTCTACTTCTGCCACTCCTCC	119
Mn-SOD	NM204211	CACTCTTCCTGACCTGCCTTACG	TTGCCAGCGCCTCTTTGTATT	146
GR	GQ853055	CTGTGGCAAAGCCCTCCTGA	ATGGGTGGGTGGCTGAAGAC	135
CAT	NM001031215	CTGTTGCTGGAGAATCTGGGTC	TGGCTATGGATGAAGGATGGAA	160
Bcl-2	NM_205339	TGTTTCTCAAACCAGACACCAA	CAGTAGGCACCTGTGAGATCG	205
Bcl-xl	NM_001025304	GAGGTACCGGAGGGCTTTCA	CAAAGCTCTGGTACGCCGTG	74
Bak-1	NM_001030920	CTGTTCGCTTCCTTCCCCTG	TTGCAGAGATGCTGTGGGAC	167
Bax	XM_422067	GGTGACAGGGATCGTCACAG	TAGGCCAGGAACAGGGTGAA	108
Cyt c	K02303.1	AGGCAAGCACAAGACTGGA	CTGACTATCACCAAGAACCACC	150
Apaf-1	XM_416167	ACCTTTCCCGTCTGGTTGTTC	AGCAATCTCTCTCCGCTTTCT	139
Casp-9	AY057940	CCAACCTGAGAGTGAGCGATT	GTACACCAGTCTGTGGGTCGG	87
AIFM1	NM_001007490	CTGGGTCCTGATGTGGGCTAT	TGTCCCTGACTGCTCTGTTGC	123
Casp-3	NM_204725	TGGCCCTCTTGAACTGAAAG	TCCACTGTCTGCTTCAATACC	139
Fas	NM_001199487	TCCACCTGCTCCTCGTCATT	GTGCAGTGTGTGTGGGAACT	78
FasL	NM_001031559	GGCATTCAGTACCGTGACCA	CCGGAAGAGCACATTGGAGT	78
FADD	XM_421073	GGGGTAAAGAGGCTGAACTCTTA	TGAGTCCTATTGCACTGCTGTC	163
Casp-8	NM_204592	GTCTCCGTTCAGGTATCTGCT	TCTCAATGAAAACGTCCGGC	143
Casp-10	XM_421936	CTGGGGGCTCCAAAAGTCC	AAAGGGGGACAAAGCCAACA	204
Bid	NM_204552	GAGCAGCTTGCTGGAGAGAA	GAGGCAGCTGGATCACAAGT	187
Grp78	NM_205491	GGTGTTGCTTGATGTGTGTCC	GCTGATTGTCAGAAGCTGTGG	134
Grp94	NM_204289	TGACCTGGATGCAAAGGTGGA	TTAAACCCCACACCATCCCTCAAC	250
CaM	NM_205005	GGAGTTGGTAAAATGAGGGAACA	ACATTGTGGACGATTGACAGTCT	233
β-actin	L08165	TGCTGTGTTCCCATCTATCG	TTGGTGACAATACCGTGTTCA	178

**Table 2:** Incidence of major histological lesions of thymus

Pathological Lesions	Time	Control group	AFB_1_ group
Congestion in red pulp	7 days	0/6	2/6
	14 days	0/6	4/6
	21 days	0/6	5/6
Nuclear fragmentation increased	7 days	0/6	1/6
	14 days	0/6	3/6
	21 days	0/6	5/6

Incidence of lesions in the thymus among animals from different experimental groups (n=6)

The ultrastructure of the thymus in the control broilers appeared normal (Figure 1E). In the AFB_1_ group, the mitochondria of some lymphocytes enlarged and contained fewer cristae, which was an indicative of mitochondrial swelling (Figure 1F). The apoptotic thymocytes could be found easily, and the hyper-condensed chromatin of apoptotic cells was under the plasma membrane, or was seen as multiple electron-dense nuclear fragments in the shape of crescent or petal (Figure 1F).

### TUNEL assay and flow cytometry analysis of apoptotic thymocytes

**Figure 2:**
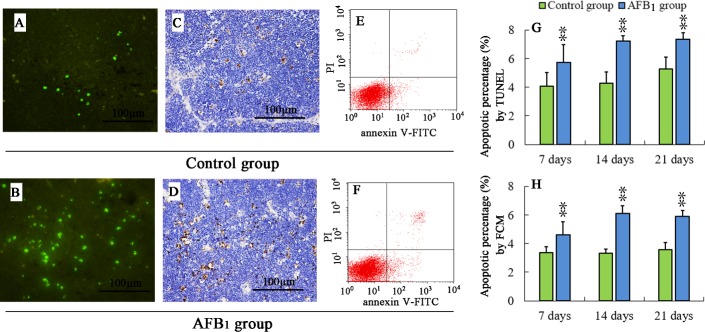
Percentage of apoptotic thymocytes from the broilers exposed to the control and AFB_1_ diets TUNEL stained slices of the thymuses from 21-day-old broilers in the control **A.**, **C.** and AFB_1_ group **B.**, **D.**. The nuclei of apoptotic cells were with green fluorescence (stained with FITC fluorescein-dUTP), or brown (stained with diaminobenzidine). Image E and F are representatives of the apoptosis by FCM. Bar graph **G.** and **H.** indicate the mean with standard deviation, and are representatives of apoptosis rate by TUNEL and FCM (*p < 0.05, **p < 0.01 *vs* control), six birds per group.

The TUNEL-positive cells were mainly around reticulocytes in cortex or around Hassall's corpuscles in medulla (Figure 2C, 2D). The apoptotic thymocytes were stained brown (Figure 2C, 2D) or with green fluorescence staining (Figure 2A, 2B). The apoptotic thymocytes in the AFB_1_ group (Figure 2B, 2D) were increased when compared with those of the control group (Figure 2A, 2C) at 7, 14 and 21 days of age. The statistical results are shown in Figure 2G.

Annexin-V-FITC was used to determine the percentage of cells undergoing apoptosis. Apoptotic cells were examined by counting the total percentage of early apoptotic cells (Annexin-V positive and PI negative) and late apoptotic cells (both Annexin-V and PI positive). The results of FCM analysis revealed similar trend as TUNEL. The percentage of apoptotic thymocytes in the AFB_1_ group (Figure 2F) was significantly higher (*p* < 0.05 or *p* < 0.01) than that in the control group (Figure 2E), and the statistical results are shown in Figure 2G at 7, 14 and 21 days of age.

### Changes of thymic T-cell subsets

**Figure 3:**
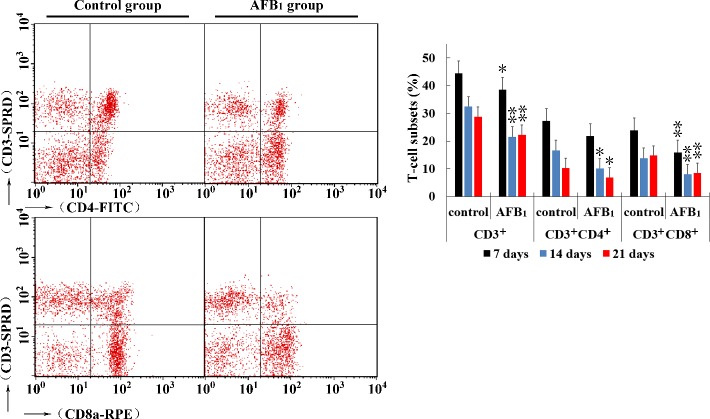
Percentages of CD3^+^, CD3^+^CD4^+^ and CD3^+^CD8^+^ T-cell in thymus from the broilers exposed to the control and AFB_1_ diets The average measured percentages of CD3^+^, CD3^+^CD4^+^ and CD3^+^CD8^+^ T lymphocytes with representative scatter diagram of the T lymphocyte subsets detected by FCM. Bar graph indicates the mean with standard deviation, and are representatives of the percentages of CD3^+^, CD3^+^CD4^+^ and CD3^+^CD8^+^ T lymphocytes of thymus (*p < 0.05, **p < 0.01 *vs* control), six birds per group.

At 7, 14 and 21 days of age, compared to the control group, the percentages of CD3^+^ and CD3^+^CD8^+^ T cells were significantly decreased (*p* < 0.05 or *p* < 0.01) in the AFB_1_ group, and significantly decreased (*p* < 0.01) percentage of CD3^+^CD4^+^ T cells was observed in the AFB_1_ group at 14 and 21 days of age. The results are shown in Figure 3.

**Figure 4:**
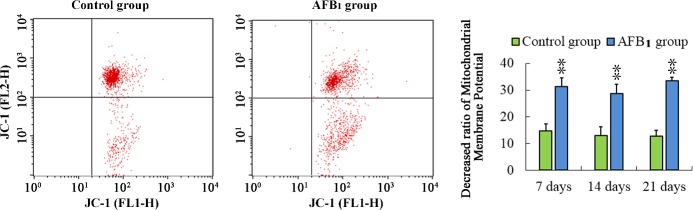
Determining mitochondrial membrane potential in thymus from the broilers exposed to the control and AFB_1_ diets Assessment of mitochondrial membrane potential in thymus from the control **A.** and AFB_1_ **B.** exposed broilers with Assessment of mitochondrial membrane potential of thymocytes with JC-1 staining by flow cytometry method. Bar graph indicates the mean with standard deviation, and are representatives of the percentage of thymocytes with lowered red fluorescence (*p < 0.05, **p < 0.01 *vs* control), six birds per group.

### Changes of Δψm

Apoptosis is frequently associated with depolarization of mitochondrial membrane potential (Δψ_m_), resulting in increased numbers of cells with reduced JC-1 fluorescence in the FL-2 channel. That is, the apoptotic population frequently presents a lower red fluorescence signal intensity. The number of cells with lower red fluorescence in the AFB_1_ group was higher than that in the control group. The results suggested that the percentage of thymocytes depolarized with collapse of the Δψm was increased (*p* < 0.01) significantly at 7, 14 and 21 days of age.

### qRT-PCR analysis of relative expressions of apoptosis- and antioxidative-related genes

**Figure 5:**
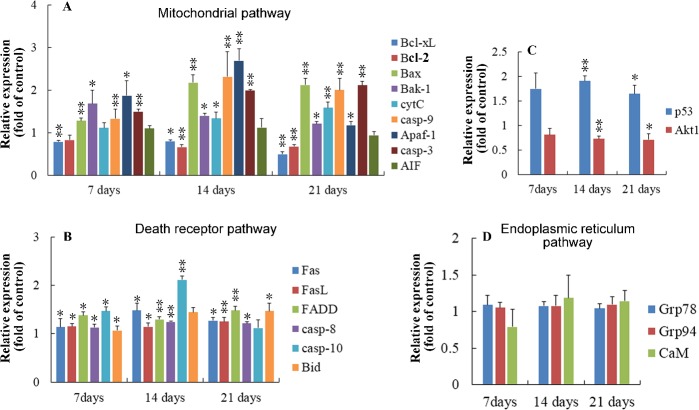
Relative expression of genes regulating apoptosis in thymus from the broilers exposed to the control and AFB_1_ diets **A.** In the mitochondrial pathway, the mRNA expressions of Bcl-xL, Bcl-2, Bax, BAak-1, cytC, casp-9, Apaf-1, casp-3 and AIF in the thymocytes of the AFB_1_-fed broilers are expressed as fold change relative to the control-fed broilers. **B.** In the death-receptor pathway, the mRNA levels of Fas, FasL, FADD, casp-8, casp-10 and Bid in the thymocytes of the AFB_1_-fed broilers are expressed as fold change relative to the control-fed broilers. **C.** The mRNA expressions of p53 and Akt1 in the thymocytes of the AFB_1_-fed broilers are expressed as fold change relative to the control-fed broilers. **D.** In the endoplasmic reticulum pathway, the mRNA expression of GRP78, GRP94 and CaM have no obvious changes compared to the control-fed broilers. All data are expressed as the mean value with deviation. *p < 0.05, *p < 0.01 *vs* control, *n* = 6 for each group.

At 7, 14 and 21 days of age, compared with the control group, the increased (*p* < 0.05 or *p* < 0.01) expressions of Bak-1, Bax, Apaf-1, caspase-3, caspase-8 and FasL were observed in the AFB_1_ group, and the mRNA level of caspase-10 increased obviously at 7 and 14 days of age. Moreover, the mRNA contents of CytC, caspase-9, Fas and Bid were significantly increased (*p* < 0.05 or *p* < 0.01) at 14 and 21 days of age. However, the expressions of Akt and Bcl-2 were significantly down-regulated at 14 and 21 days of age. What's more, the mRNA content of Bcl-xL was significantly lower than those in the control group at 7, 14 and 21 days of age. There were no significant changes observed in the expressions of AIF, Grp78, Grp94, and CaM. The results were shown in Figure 5.

**Figure 6:**
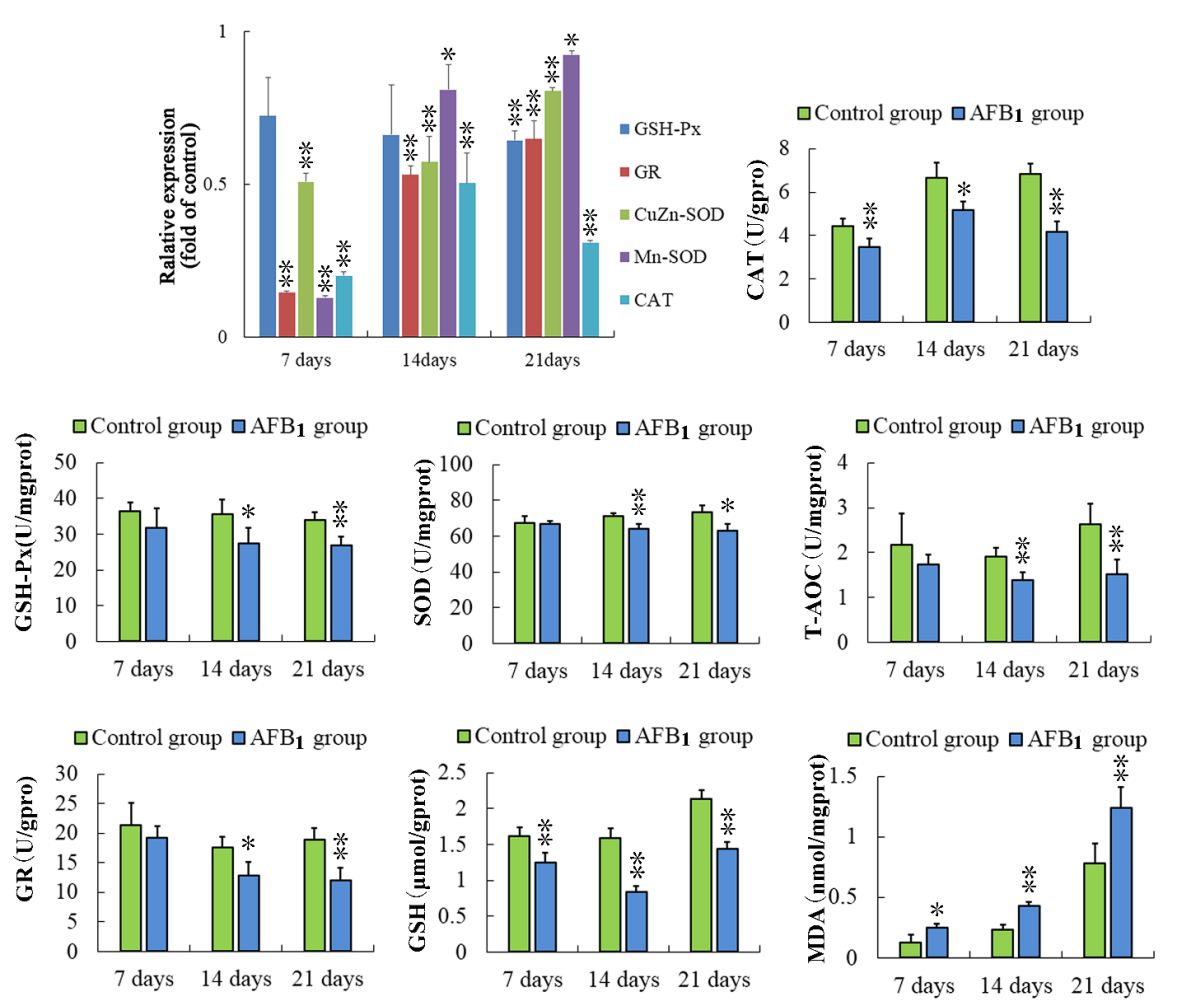
Assessment of the antioxidant function in thymus from the broilers exposed to the control and AFB_1_ diets The mRNA expressions of GSH-Px, GR, CuZn-SOD, Mn-SOD, and CAT in the thymocytes of the AFB_1_-fed broilers are expressed as fold change relative to the control-fed broilers. Others bar graph indicate the mean with standard deviation, and are representatives of the enzymatic activities of GSH-Px, GR, CuZn-SOD, Mn-SOD, CAT and T-AOC, and the contents of GSH and MDA (*p < 0.05, **p < 0.01 *vs* control), six birds per group.

Comparing with those of the control group, the expressions of glutathione reductase (GR), CuZn-superoxide dismutase (CuZn-SOD), Mn-superoxide dismutase (Mn-SOD) and catalase (CAT) were significantly decreased (*p* < 0.05 or *p* < 0.01) in the AFB_1_ group at 7, 14 and 21days of age. What's more, the mRNA contents of glutathione peroxidase (GSH-Px) were significantly lower at 21 days of age than that in the control group. (Figure 6)

### Antioxidation biochemistry analysis parameters

At 7, 14 and 21 days of age, the activities of SOD, CAT, GSH-Px and GR were significantly lower (*p* < 0.01 or p < 0.05) than those in the control group. Comparing to the control group, the decreased (*p* < 0.05 or *p* < 001) contents of GSH and increased (*p* < 0.05 or *p* < 0.01) contents of malonic dialdehyde (MDA) were observed in the AFB_1_ group at 7, 14 and 21 days of age. Meanwhile, the total ant-oxidative capacity (T-AOC) was significantly decreased (*p* < 0.01) in the AFB_1_ group at 14 and 21 days of age. The results are shown in Figure 6.

## DISCUSSION

AFB_1_ exhibits toxic effects in humans as well as in all animal species so far investigated [26]. Broilers are sensitive to AFB_1_, and a low dosage of AFB_1_ can induce immunosuppression, which showed as the decrease of T cell subsets, some cytokines contents, antibody titers, and complement activity [27-30]. In the present study, the toxic effects of AFB_1_ on thymus were investigated in chicken. As revealed by histopathological examination, T-cell subsets assay and apoptosis analysis, an obvious congestion in medulla (Figure 1B) and increase of nuclear debris in cortex (Figure 1D), significant decrease of CD3^+^, CD3^+^CD4^+^ and CD3^+^CD8^+^ T-cell percentages (Figure 3), and increase of apoptotic thymocytes (by flow cytometer and TUNEL essay) (Figure 2) were consistently observed in the AFB_1_ group, which were in line with our earlier research [31]. Our previous and present studies both demonstrated that the apoptosis of thymocytes might be closely related to AFB_1_-induced immunosuppression, but it should be further clarified that if mitochondria, death receptor and endoplasmic reticulum mediated apoptotic pathways were all involved in this apoptotic procedure. Our study is the first to answer this question by determining 18 key signaling molecules in the three apoptotic pathways by using a broiler model.

Apoptosis is an energy-dependent process of cell suicide in response to a variety of stimuli and is characterized by a number of distinct morphological features including cell shrinkage, plasma membrane blebbing, chromatin condensation, and ultimately, cell fragmentation into apoptotic bodies which are phagocytosed without provoking an inflammatory response [32, 33]. In the AFB_1_ group, the apoptotic lymphocytes with condensed chromatin could be easily found, and obvious swollen mitochondria with degenerated cristae was observed in the thymic lymphocytes. These ultrastructural changes mean that the apoptotic mechanisms maybe associated with mitochondria signaling pathway. Because depolarization of the Δψ_m_ was observed to occur early during mitochondria mediated apoptosis [34], we detected the changes of Δψ_m_ by JC-1 staining method. The result showed that the percentage of thymocytes with low red fluorescence (thymocytes with depolarized Δψ_m_) was increased in the AFB_1_ group, suggesting that mitochondrial pathway was activated during the AFB_1_ induced apoptosis in thymocytes.

Based on the increase of Bax and caspase-3 mRNA expression, and the decrease of Bcl-2 mRNA expression, it has been suggested that the AFB_1_-induced apoptosis could be triggered through mitochondria pathway in the thymocytes, splenocytes and bursa of Fabricius cells of chicken [15], and in the hepatocytes of duckling [35]. In our study, when chickens exposed to AFB_1_, the decreased expressions of Bcl-2 and Bcl-xL, increased expressions of Bax, Bak, cytC, apaf-1, caspase-9 and caspase-3 confirmed that the activated mitochondrial pathway was important for AFB_1_ induced thymocytes’ apoptosis in chicken. The mitochondrial pathway can be regulated by the pro- and anti-apoptotic Bcl-2 family proteins, including Bax, Bak, Bcl-xL and Bcl-2 [36], as well as caspase family proteins, such as caspase-9, caspase-3, and caspase-7 et al. [37]. The down-regulation of Bcl-xL, Bcl-2 and up-regulation of Bak, Bax can induce increased permeabilization of mitochondrial outer membrane, thereby causing release of cytC, apoptosis-inducing factor (AIF), and ATP from mitochondria [38]. Subsequently, the formation of tetramer (composed of caspase-9, Apaf-1, cytC and dATP) leads Apaf-1 apoptosome to facilitate the autocatalytic activation of caspase-9 [39]. And then the activated caspase-9 in turn cleaves and activates effector caspases (caspase-3, caspase-7 et al) [40]. In addition, the down-regulated expression of Bcl-2 and Bcl-xL might trigger PI3K/Akt pathway and excessive expression of Bid [41]. Moreover, p53 could participate in apoptosis through mitochondrial pathway by up-regulating the expression of Bax and down-regulating the expression of Bcl-2 [42, 43]. Collectively, our results revealed that mitochondria mediated apoptotic pathway was involved in AFB_1_ induced thymocytes apoptosis.

The death receptor pathway can be induced through the activation of death receptors, such as Fas, which requires binding to the Fas ligand (FasL) [44]. And then caspase-8 is activated through the Fas associated death domain (FADD)[44]. Josse et al. [45] observed that AFB_1_ at a concentration of 0.05 μM could cause an higher expression of Fas in human hepatocytes. In the present study, AFB_1_ administration led to the increased expression of Fas, FasL, FADD, caspase-8, caspase-10, and Bid in the thymus. Our results demonstrated that the excessive thymocytes apoptosis induced by AFB_1_ involved the death receptor pathway. According to previous studies, caspase-8 activates not only downstream of caspase-3 and caspase-7 [46], but the Bcl-2 homology Bid, which translocates to mitochondria and blinds to Bax, enable a crosstalk to the mitochondrial apoptotic pathway [47].

The endoplasmic reticulum pathway is initiated by endoplasmic reticulum stress due to a number of factors, including cytotoxicity, and nutrient limitation [48]. Grp78 and Grp94 function as molecular chaperones and can bind to malfolded proteins and unassembled complexes [49, 50]. CaM, a highly conserved Ca^2+^ binding protein, is also related to endoplasmic reticulum pathway [51]. In the present study, no changes were observed in the expressions of GRP78, GRP94 and CaM in the AFB1 group when compared with those of the control group. It is evident that the endoplasmic reticulum pathway may not involve in AFB_1_ induced thymocytes apoptosis in this experiment. However, it is should be further clarified in the future studies if prolonged or excessive exposure of AFB_1_ could active this pathway in thymus.

AFB_1_ is bioactivated by P450 enzymes to generate AFB1exo-8,9-epoxide, which can react with DNA, forming trans-8,9-dihydro-8-(N^7^-guanyl)-9-hydroxyaflatoxin B_1_ (AFB_1_-N7-Gua) [52]. The DNA adducts provoke severe steric alterations in DNA that impair DNA-dependent metabolic process, including DNA replication and transcription [53]. It is well accepted that oxidative stress is an apoptosis inducer [54]. Previous studies showed that an imbalance between reactive oxygen species (ROS) and the antioxidant reserve may cause oxidative stress, which could result in DNA damage and mitochondrial lesions, and trigger apoptosis [17, 55, 56]. In the current study, the decreased activities of GSH-Px, SOD, CAT, GR, T-AOC, increased concentration of MDA, decreased content of GSH and decreased mRNA levels of antioxidant enzymes (GSH-Px, SOD, CAT and GR), were found in broilers fed with AFB_1_ diet (Figure 6). These results suggested that the intake of AFB_1_ could induce accumulation of ROS through inhibiting the production and activity of antioxidant enzymes. Clearly, our results demonstrated that oxidative stress may be a main mediator of AFB_1_ induced excessive apoptosis of thymocytes in chickens.

**Figure 7:**
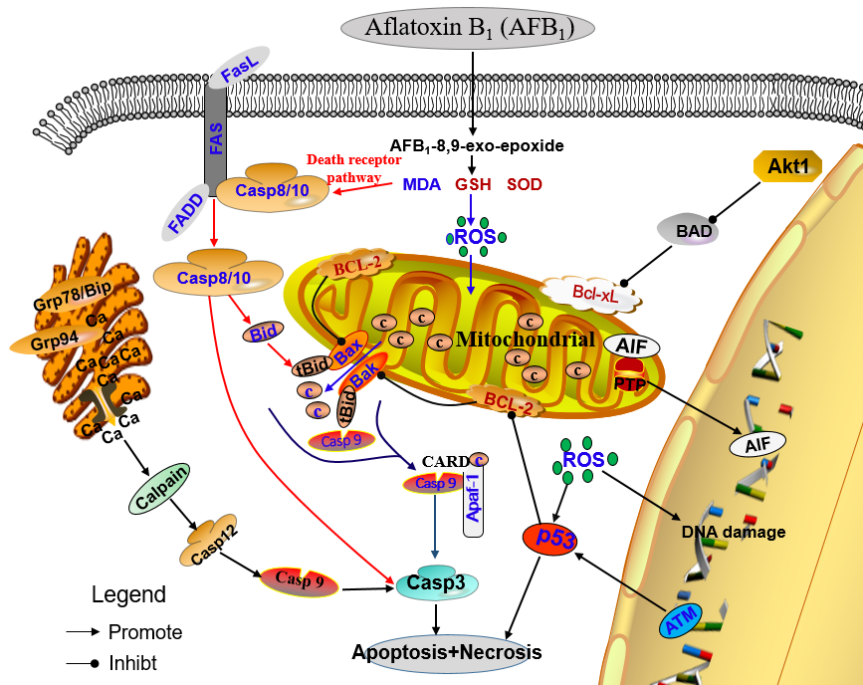
Schematic diagram of the proposed mechanisms of aflatoxin B_1_ induced apoptosis

In summary, our study shows that dietary AFB_1_ exposure is able to induce excessive apoptosis of thymocytes in chicken by triggering mitochondrial and death receptor mediated apoptosis pathways (Figure 7). We also demonstrate that the mechanism of oxidative DNA damage is involved in the apoptosis. Future studies will focus on a deeper understanding of the mechanisms of AFB1-induced immunosuppression.

## MATERIALS AND METHODS

Investigation has been conducted in accordance with the ethical standards and according to the Declaration of Helsinki and according to national and international guidelines and has been approved by Sichuan Agricultural University Animal Care and Use Committee (Approval No: 2012-024).

### Chickens and diets

One hundred and fifty-six one-day-old healthy Cobb broilers were purchased from Chia Tai Group (Wenjiang, Sichuan, China), and were randomly divided into two equal groups of three replicates and 26 birds per replicate, namely control group (0 mg/kg AFB_1_) and AFB_1_ group (0.6 mg/kg AFB_1_). The basal diet, namely the control diet, was formulated according to National Research Council (NRC, 1994) [57] and Chinese Feeding Standard of Chicken (NY/T33-2004) recommendations. The AFB_1_-contaminated diet was made, referring to the method described by Kaoud [58]. Briefly, 27 mg AFB_1_ (A6636, Sigma-Aldrich, USA) was dissolved into 30 ml methanol, and then the 30 ml mixture was mixed into 45 kg corn-soybean basal diet to formulate the AFB_1_-contaminated diet. The equivalent methanol was mixed into corn-soybean basal diet to produce the control diet. Then the methanol of diets was evaporated at 98 °F (37°C). The AFB_1_ concentrations were analyzed by HPLC (Waters, Milford, MA, USA) with fluorescence detection (Waters, Model 2475, Milford, MA, USA), and the AFB_1_ concentration were determined as < 0.001mg/kg and 0.061mg/kg respectively in the control diet and AFB_1_ diet. Broilers were housed in cages with electrically heated units and provided with water as well as aforementioned diet ad libitum for 21 days.

### Histopathological and ultrastructural examination

Six broilers in each group were euthanized at 7, 14 and 21 days of age. The thymuses were fixed in 4% paraformaldehyde (PFA) and routinely processed in paraffin. Thin sections (5 μm) of each tissue were sliced and mounted on glass. Slides were stained with hematoxylin and eosin Y. The histological structures of the tissues were observed and photographed with a digital camera (Nikon, eclipse 50i, Japan).

At the end of the trial, one chick per replicate in each group was euthanized and then immediately necropsied. Small pieces of thymus tissues were rapidly fixed with 2.5% glutaraldehyde and post-fixed in 2% Veronal acetate-buffered OsO_4_. After dehydration in graded alcohol, the tissues were embedded in Araldite. The blocks were sectioned in a microtome with a glass knife. Sections, 65-75 nm thick, were placed in uncoated copper grids. The sections were stained with uranyl acetate, and post-stained with 0.2% lead citrate. The subcellular structure of thymus was examined with a Hitachi H-600 electron microscope (Japan).

### Determination of the thymic T-cell subsets

The thymuses of six birds in each group were sampled to determine the T cells subsets by flow cytometry method at 7, 14 and 21 days of age. Thymic cell suspension was prepared by gently cutting with scissors, and filtered through a 300-mesh nylon screen. Then the cells were washed and suspended in phosphate buffer (PBS, PH: 7.2) at a concentration of 1×10^6^ cells/mL. 100 μL cell suspension was transferred to another centrifuge tube, and stained with mouse anti-chicken CD3-SPRD (Cat. No: 8200-13, Southern Biotech, USA), mouse anti-chicken CD4-FITC (Cat. No: 8210-02, Southern Biotech, USA), and mouse anti-chicken CD8a-RPE (Cat. No: 8220-09, Southern Biotech, USA) for 15min at 4°C in the dark. After 2 mL PBS added and centrifugal elutriation performed once, the supernatant was discarded. The cells were resuspended in 450μL PBS and determined by BD FACSCalibur flow cytometer (FCM. BD. USA).

### Annexin V-FITC/PI double staining assay

The percentage of apoptotic cells was determined by flow cytometry. 5 μL of Annexin V-Fluorescein isothiocyanate (V-FITC) (BD Pharmingen, USA, 51-65874X) and 5 μL of Propidium iodide (PI) (BD Pharmingen, USA, 51-66211E) were added into 100 μL cell suspension (made in procedure T cell subsets determination), and incubated at 25°C in the dark for 15 min. 450 μL of 1×Annexin binding buffer (BD Pharmingen, USA, 51-66121E) was added to the mixture, and the percentages of apoptotic cells were assayed by FCM within 1 hour.

### TUNEL assay

The DNA fragmentation indicative of apoptosis was examined using terminal deoxynucleotidyl transferase-mediated dUTP nick end labeling method (TUNEL). TUNEL assay was performed using Insitu Cell Death Detection Kit (Cat. NO. 11684817910, Roche Molecular Biochemicals, Germany) according to the instructions of the manufacturer, as described by Tayman [59].

### Detection of mitochondrial membrane potential (Δψm)

JC-1 (Cat.No.551302, BD, USA) was used to determine mitochondrial membrane potential (Δψm). A total of 1 ml cell suspension (made in procedure T cell subsets determination) was transferred into 5 mL culture tube and centrifuged. Afterwards, 0.5 mL of JC-1 working solution was immediately added and gently mixed. And then incubating the mixture for 15 min at 37°C under 5% CO_2_ incubator. At the end of the incubations, cells were washed twice with 1× Assay Buffer cells. And then resuspended each pellet in 450 μL 1× Assay Buffer. And then Δψm was assayed by FCM within 30 minutes.

### Quantitative real-time PCR (qRT-PCR)

At 7, 14, and 21 days of age, thymuses from six birds in each group were removed and immediately stored in liquid nitrogen. Then, thymuses were homogenized by crushing with a mortar and pestle. The powdered tissues were collected into eppendorf tubes and stored at −80°C. Total RNA was extracted from thymus using TriPure Isolation Reagent (Cat No. 11667165001, Roche Applied Science, Germany) following the procedure provided by manufacturer. The yield of extraction was assessed by measuring light absorbency at 260 nm, and the quality of RNA was detected by calculating the ratio of the absorbency at 260 and 280 nm. Extracted RNA immediately reverse-transcribed into cDNA by using Transcriptor First Strand cDNA Synthesis Kit (Cat No: 04897030001, Roche Applied Science, Germany), according to the manufacturer's instructions. And then the cDNA was used as a template for quantitative real-time PCR analysis.

For qRT-PCR reactions, 20 μL mixtures were made by using FastStart Universal SYBR Green Master mix (Cat No: 04913914001, Roche Applied Science, Germany) containing 10 μL faststart universal SYBR green master (ROX), 0.6μL forward primer, 0.6 μL reverse primer, 6.8 μL RNAase-free water and 2 μL cDNA. Reaction conditions were set to 10 min at 95°C (first segment, one cycle), 10 s at 95°C and 30 s at melting temperature (T_m_) of a specific primer pair (second segment, 44 cycles) followed by 10 s at 95°C, and 72°C for 10 s (dissociation curve segment) using Thermal Cycler (Step One Plus, Applied BioSystems, USA). Gene expression was analyzed, and β-actin was used as an internal control [60, 61]. Accession number list in Table 1 was obtained from GenBank of NCBI. Primers were designed with Primer 5 and synthesized by Sangon Biotech (Shanghai, China) (Table 1). The qRT-PCR data were analyzed with 2^−ΔΔCt^ calculation method described by Livak and Schmittgen [62].

### Biochemical analysis

At 7, 14 and 21 days of age, six broilers in each group were euthanized and immediately necropsied. Then thymuses were immediately removed and put into 0°C 0.85% NaCl solution. 1 g thymus was homogenized with 9 mL 0.85% NaCl solution. After the homogenates were centrifuged at 3500×g at 4°C, the total protein in the supernatant was determined by total protein quantification kit (A045-2). The activities of SOD (A001-1), CAT (A007), GSH-Px (A005) and GR (A062), contents of GSH (A006) and MDA (A003-2), and T-AOC (A015) in the supernatant were detected using commercial kits (NJJCBIO, Nanjing, China), according to the manufacturer's instructions.

### Statistical analysis

The significance of difference between two groups was analyzed by variance analysis, and results are expressed as the mean value with deviation. The analysis was performed using the independent sample *t* test of SPSS software for Mac v.20.0 (IBM Corp, Armonk, NY, USA) and a value of *p* < 0.05 was considered significant, while *p* value < 0.01 was considered markedly significant.
